# Prediction of Pasta Colour Considering Traits Involved in Colour Expression of Durum Wheat Semolina

**DOI:** 10.3390/foods14030392

**Published:** 2025-01-24

**Authors:** Antonio Troccoli, Donatella Bianca Maria Ficco, Cristiano Platani, Maria Grazia D’Egidio, Grazia Maria Borrelli

**Affiliations:** 1 Consiglio per la Ricerca in Agricoltura e l’Analisi dell’Economia Agraria, Centro di Ricerca Cerealicoltura e Colture Industriali, S.S. 673, m 25200, 71122 Foggia, Italy; antonio.troccoli@crea.gov.it (A.T.); donatellabm.ficco@crea.gov.it (D.B.M.F.); 2 Consiglio per la Ricerca in Agricoltura e l’Analisi dell’Economia Agraria, Centro di Ricerca Orticoltura e Florovivaismo, Località Stella, Via Salaria, 1, 63030 Monsampolo del Tronto, Italy; cristiano.platani@crea.gov.it; 3 Consiglio per la Ricerca in Agricoltura e l’Analisi dell’Economia Agraria, Centro di Ricerca Ingegneria e Trasformazioni Agroalimentari, Via Manziana, 30, 00189 Roma, Italy; mariagrazia.degidio@gmail.com

**Keywords:** durum wheat genotypes, environment, oxidative enzymes, carotenoid pigments, colorimetric indices, pasta, high-performance index

## Abstract

Colour plays an important role among the quality traits of durum wheat, attracting consumer attention for the pasta market. The traits involved in colour expression are affected by genotype, environment, and processing. In the present study, based on eighteen durum wheat genotypes grown in eight environments, the effects of different traits related to colour expression were evaluated. Carotenoid pigments, such as lutein and β-carotene content; yellow and brown indices; and lipoxygenase, peroxidase, and polyphenoloxidase activities were analysed in semolina. The effects of processing were evaluated by measuring both the content of carotenoid pigments and colorimetric indices in pasta. The genotype, the environment, and their interaction were significant for all traits, although with a strong prevalence of genotypic effects, except for the brown index. After processing, a decrease in carotenoid content and the yellow index (86.7% and 16.0%, respectively) was observed, while the brown index increased (8.2%). A multiple regression analysis was performed on semolina traits, and the yellow index emerged as the main predictor for pasta colour, strengthening this trait as a fast and reliable criterion of selection. A High-Performance Index tool was also used to identify the genotype and environment that better combine all traits, positively influencing colour expression. All this information can be used in durum wheat breeding programmes for the prediction of pasta colour.

## 1. Introduction

Durum wheat (*Triticum turgidum* L. ssp. *durum*) represents an important component of the Mediterranean diet. It is the tenth most cultivated cereal in the world, and among wheats, it is the second most produced crop after bread wheat [1]. Durum wheat covers about 13.7 million ha, representing approximately less than 7% of the total global wheat cultivation area, with a global production of 34.3 million tons, considering the average values of 2018–2022 [2]. It is the main crop in many areas of the Mediterranean basin, whose countries account for almost 50% of the cultivated area and global production of durum wheat [3]. Mediterranean countries are also the main consumers of products derived from durum wheat, such as pasta, bread, bulgur, and couscous. Currently, Canada is the leading producer of durum wheat, followed by Italy, Algeria, and Turkey [1,4], and it is the largest durum wheat exporter in the world, with its main destinations being Algeria, Italy, and Morocco [1,5].

Ensuring durum wheat quality is essential to maintaining competitiveness in the market. Consumer choices play an important role in directing breeding towards the improvement of traits linked to the quality of durum wheat end products. Among these end products, pasta represents the most common and popular staple cereal food thanks to its sensory and nutritional properties [6,7], and it is the most widespread cereal food in Italian cuisine. Its quality is the complex result of the influence of different factors which intervene in various stages of the durum wheat supply chain [8]. Quality is mainly influenced by genotype but also by the environment (weather and soil), crop management, and the primary (milling) and secondary (pasta making) processing steps [9,10].

Oxidative properties that are associated with colour are particularly important for durum wheat commercial value, since consumers prefer semolina and pasta with a bright yellow colour. Colour is influenced by the interaction between a yellow component and a brown one, both resulting from the balance between the oxidative and antioxidative components of grain.

The yellow component is mainly affected by (i) the endogenous content of carotenoid pigments in kernels, antioxidant compounds that act as scavengers of free radicals, providing significant health benefits; (ii) their residual content after milling; and (iii) their degradation that occurs during pasta processing due to oxidative enzymes, especially lipoxygenase (LOX) and, to a minor extent, peroxidase (POD) [11,12,13,14]. Among the carotenoid pigments, lutein and, to a minor extent, β-carotene, which is an important precursor of vitamin A in mammalians, are mainly present in durum wheat grain [15].

The brown component is the result of the oxidation of compounds such as polyphenols, which occurs in the endosperm, during kernel maturation, and in semolina and dough during processing, due to enzymes such as peroxidase (POD) and polyphenoloxidase (PPO), and it is also a result of the ash content, an indicator of residual bran fraction [16,17,18,19,20].

Genotype, environment, and their interaction affect all the cited traits in grain and semolina to a different extent [11,21,22,23,24,25], together with physical and chemical variations that occur during milling and pasta processing [11,12].

Two approaches were used in the present study to evaluate the colour traits of durum wheat products. Firstly, the components of the variance for genotype, environment, and their interaction were estimated in terms of the colour-related traits of semolina and pasta in a set of eighteen durum wheat genotypes grown throughout the Italian territory in eight different climate areas. Secondly, the semolina trait that could be considered as the best predictor of the final pasta colour was identified by multiple regression analysis.

Finally, to identify the genotype and environment that best combine the colour traits studied, a High-Performance Index (HPI) tool was used. This is a mathematical tool employed to simultaneously evaluate, in a simple and objective way, multiple traits that are different in their units of measurement and also have opposite effects [26]. The use of this tool allows us to simplify the interpretation of data, and its application can be easily extended to other studies by adapting the procedure to the specific objective.

## 2. Materials and Methods

### 2.1. Plant Materials

Eighteen varieties of durum wheat, randomly selected from those mainly released in the past 90 years, were grown in the 1999–2000 growing season throughout the Italian territory in eight environments with different agro-climatic characteristics (Table S1), included in the Italian National Cereal Test Network. The experimental design was a lattice square with three completely randomized replicates. The plots were 10 m^2^ with eight rows, spaced 17 cm apart. The cultivation of durum wheat varieties was carried out in different locations within each agro-climatic environment using standardized cultural practices. For each variety, equal amounts of grain samples collected from the different locations of the same environment were mixed to obtain a composite sample [27]. The appropriateness of this standardized sampling procedure, usually applied in the Italian National Cereal Test Network, was assured by the small genotype × location interaction for the main quality traits analysed [28]. The detailed thermo-pluviometric trend for all climate areas was described in Desiderio et al. [29].

**Figure 1:**
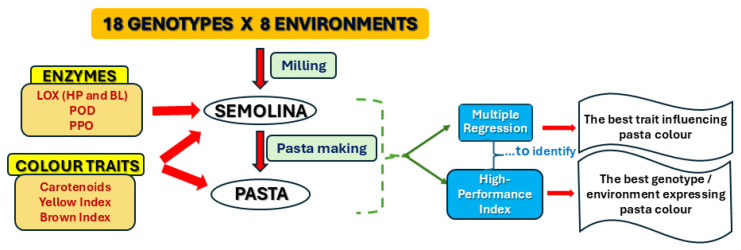
Experimental process.

A flow chart showing the entire experimental process is displayed in Figure 1.

### 2.2. Semolina Production

Cleaned seeds were conditioned overnight to 16.5% moisture and processed by an MLU 202 experimental mill (Bühler Brothers, Uzwill, Switzerland). The extraction rate was on average 65%. Semolina was stored at 4 °C up to analysis.

### 2.3. Pasta Processing

Semolina was processed into spaghetti (1.7 mm diameter) using a 2 kg capacity laboratory plant (NAMAD, Rome, Italy). A low-temperature drying procedure (50 °C for 20 h) was applied in the pilot plant (AFREM, Lyon, France). Dried pasta, finely ground by a Tecator Cyclotec 1093 laboratory mill (International PBI, Milano, Italy) and sieved (<500 μm), was stored at 4 °C up to analysis.

### 2.4. Enzyme Assays in Semolina

Semolina’s hydroperoxidation and bleaching activities of LOX, POD, and PPO activities were determined in triplicate and expressed as μmoles of changed substrate min^−1^g^−1^. Crude extracts for LOX and POD analysis were obtained as described by Borrelli et al. [12], whereas those for PPO analysis were obtained according to González et al. [30]. The protein content of extracts was evaluated by the Lowry et al. [31] method, using crystalline bovine serum albumin as a standard.

#### 2.4.1. Hydroperoxidation and Bleaching Activities of LOX

Both LOX activities were evaluated using a λ18 UV/vis Spectrometer (Perkin Elmer, Norwalk, CT, USA), equipped with a water-jacketed cell holder. The optimal pH for both activities was chosen according to Barone et al. [32].

LOX hydroperoxidation activity (Hp) was determined at 25 °C and at pH 6.6 by measuring conjugate diene absorption at 234 nm, according to Borrelli et al. [12]. One unit of enzymatic activity corresponded to the production of 1 μmol of conjugate hydroperoxydienoic min^−1^, using a molar extinction coefficient of 28 mM cm^−1^.

The β-carotene bleaching activity (BL) of LOX was evaluated at 25 °C and at pH 5.2 by measuring the decrease in absorbance at 460 nm, as described by Borrelli et al. [12]. One unit of enzyme activity corresponded to the destruction of 1 μM of β-carotene min^−1^, using a molar extinction coefficient of 123.5 mM cm^−1^.

#### 2.4.2. Peroxidase Activity

Peroxidase (POD) activity was determined at 30 °C by measuring the slope from the linear increase in absorbance at 470 nm at pH 4.2, due to the oxidation of guaiacol to tatraidroguaiacol, in the presence of hydroxide peroxide, using a λ20 UV/vis Spectrometer (Perkin Elmer), as described by Borrelli et al. [12]. One unit of enzyme activity was defined as the change in one absorbance unit per min with an ε value of 26.6 mM^−1^.cm^−1^.

#### 2.4.3. Polyphenoloxidase Activity

Polyphenoloxidase (PPO) activity was evaluated at 25 °C by measuring the increase in absorbance at 480 nm, as a result of DOPA conversion into dopachrome, according to Okot-Kotber et al. [33], with little modifications. The reaction mixture (3 mL) contained 10 mM L-DOPA in 0.1 M phosphate buffer, pH 6.5, and a suitable amount of extract to give a linear initial velocity of the reaction. One unit of PPO activity was defined as the amount of enzyme resulting in a change in absorbance of 0.001 min^−1^. PPO activity was discriminated from that of POD using tropolone as a selective inhibitor for PPO.

### 2.5. Laboratory Analyses of Semolina and Pasta

#### 2.5.1. Determination of β-Carotene and Lutein Content

β-carotene and lutein contents were measured by HPLC with an isocratic solvent programme, using an Agilent HPLC system 1100 (Agilent Technologies, Waldbronn, Germany), and expressed as μg/g, dry matter (DM), according to Borrelli et al. [12]. Analyses were carried out in duplicate, and quantification was performed with reference to a calibration curve obtained using relative standards.

#### 2.5.2. Yellow and Brown Indices

A chromameter (CR200, Minolta, Osaka, Japan) was used to determine semolina and pasta yellow (b*) (YI) and brown (100-L*) (BI) indices, according to CIELAB colour space system [34] which measures lightness (L*: 100 white, 0 black), redness (a*: +60 red, −60 green), and yellowness (b*: +60 yellow, −60 blue) values. Each index was the average of three measurements.

### 2.6. Procedure for Calculating High-Performance Index (HPI)

To identify superior genotypes which better express traits influencing the colour of durum wheat products, the HPI was used. This descriptive tool is a dimensionless index derived from a mathematical algorithm developed in Microsoft Excel by Troccoli et al. [26]. The algorithm requests some constraints on analytical variables to be specified: in particular, the optimal condition consists of low LOX, POD, and PPO activities in semolina and a high content of pigments and the YI, as well as a low BI, in semolina and pasta. Starting from all the analytical variables detected in semolina and pasta, the constraints imposed on each of them are used by the mathematical algorithm to firstly calculate the standardized deviations from the overall mean of each variable and then to assign a positive or negative score that increases (up to a maximum of 2) as the size of the deviation increases, obtaining the final value of the HPI of each genotype by adding all the scores, according to the detailed procedure described in Troccoli et al. [26]. Considering that each variable comes from eight environments, the absolute maximum HPI score that each genotype can achieve is “2 × *n* variables × *m* environments” (2 × 12 × 8= 192). The same approach was applied considering the environment as a categorical variable. The HPI is graphically represented with a spider chart, sorting the data in descending order. The chart is composed of axes that extend from a central point, each of which represents the HPI value for every genotype.

### 2.7. Statistical Analysis

ANOVA-Type II and variance component analysis were performed considering both environment and genotype as random factors.

The mean values of genotype x environment interaction traits were used to obtain a Pearson correlation matrix and the *p*-values of the correlation coefficient.

A multiple regression analysis was performed on all semolina traits used as the predictors (independent variables) of the yellow index of pasta (dependent variable). Mallow’s Cp statistics were used as a criterion to find the best regression model involving a subset of predictor variables. The significance of the regression model was tested with an ANOVA. A collinearity test and other statistical parameter calculations were performed on the predictor variables included in the regression model. The Durbin–Watson statistic test was conducted on the residuals to exclude the autocorrelation phenomenon.

Based on the simple correlation matrix between all traits analysed in the eighteen genotypes, a Principal Component Analysis (PCA) was performed using the mean genotypic values.

Statistical analyses were performed using the STATISTICA program (StatSoft Italia srl, vers. 8.0, 2007). PCA was performed using PAST software 4.17c (http://palaeo-electronica.org/2001_1/past/issue1_01.htm, accessed on 8 January 2025).

*Chemicals:* Linoleic acid, β-carotene, carotenoid standards, Tween 20, DOPA, tropolone, and LOX soybean (type IV) were purchased from Sigma Aldrich Chemical Co. (St. Louis, MO, USA). Reagents for HPLC and gas chromatography were purchased from J.T. Baker (Deventer, Holland). Tween 80 was purchased from Merck (Darmstadt, Germany).

## 3. Results and Discussion

### 3.1. Contribution of Genotype, Environment, and Their Interaction to Colour-Related Traits in Semolina and Pasta

Among quality traits, the colour of the end products, semolina and pasta, plays an important role both for aesthetic aspects, widely appreciated by consumers, and for nutritional implications, mainly linked to the presence of carotenoid pigments.

The importance of genotype, environment, and their interaction in terms of the traits directly and indirectly related to semolina colour was evaluated by ANOVA-Type II (Table 1).

**Figure 2:**
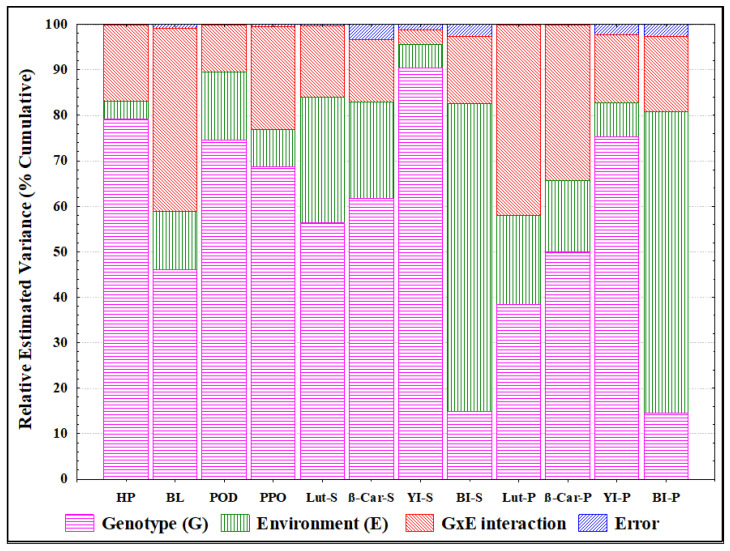
Relative variance component, expressed in percentage of total variance, for all traits evaluated in semolina and pasta of eighteen genotypes grown in eight different Italian environments. Columns indicate cumulative sums of components. HP = hydroperoxidation activity of LOX; BL = bleaching activity of LOX; POD = peroxidase activity; PPO = polyphenoloxidase activity; Lut = lutein; β-Car = β-carotene; YI = yellow index; BI = brown index; S = semolina; P = pasta.

The genotype, the environment, and their interaction were highly significant for all traits (Table 1). To estimate the percentage of total variance due to environment, genotype, and their interaction on the analysed traits, a variance component analysis was performed. Excluding BL, BI-S, Lut-P, and BI-P, most traits were strongly influenced by genotype, ranging from 50% (β-Car-P) to 90% (YI-S) of the total variance (Figure 2; Table S2). According to Schulthess et al. [35], the environment played an important role (above 65%) only for the BI-S and BI-P traits, while a moderate effect ranging from 4% (HP) to 21% (β-Car-S) on the remaining traits was found (Figure 1; Table S2). This effect could be explained with the correlation between the BI and ash content, which is significantly influenced by the environment [8,36,37]. The minor effects of the environment on traits related to the yellow colour component are in agreement with Sisson et al. [38].

The effects of the G x E interaction were quite evident on Lut-P (42%), the BL activity of LOX (40%), and β-Car-P (34%) traits but less impactful on the remaining traits. The error component of total variance on all traits can be considered completely marginal.

Carotenoid pigment content is a quantitative character regulated by various genes with additive effects and a minor effect of the environment [39]. The results of our study confirmed the main effects of genotype on yellow pigment and enzyme activities in semolina. These results agree with previous studies [14,21,22,23,24,25,40,41], although some of them evidenced a marginal effect of environmental conditions, including year, location, agronomic practices, and their interaction [40,41,42,43].

POD and PPO showed great variability among genotypes with respect to HP and BL (Table 1). The lowest HP values were found in Saadi, Parsifal, and Creso, while the highest ones were in Preco, Torrebianca, Duilio, and Simeto. Semolina in Simeto, in addition to having the highest POD and PPO values, had a very negative outcome in terms of its final colour (Table 1). In general, in semolina, the lutein and β-carotene content and the YI were high in Preco and Duprì and low in Gianni, whereas the BI was the highest in Simeto and the lowest in Parsifal and Gianni. This trend was also generally found in pasta. The increase in carotenoid content in newer varieties with respect to older ones (1.194 vs. 0.793 µg g^−1^, db, on average) confirmed the positive results of breeding programmes aimed at improving the yellow colour of durum wheat due to the high value of the heritability of carotenoid pigment content [21,24,25,42].

### 3.2. Effects of Processing on Pasta Colour

#### 3.2.1. Variation in Colour-Related Traits in Pasta

**Figure 3:**
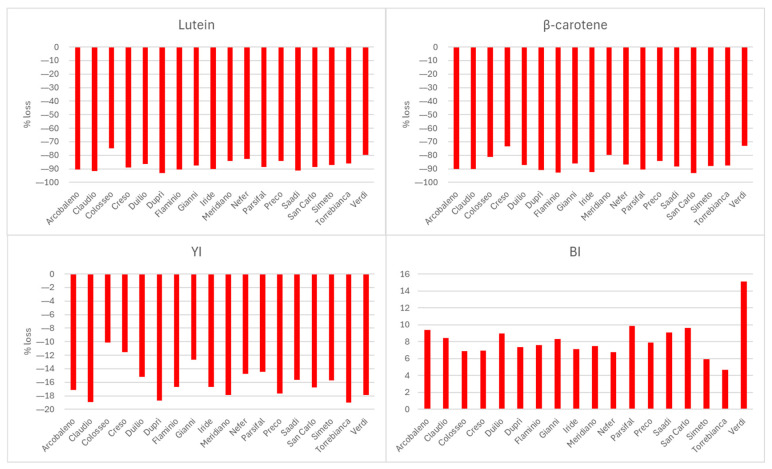
Percentage variation in colour traits in eighteen genotypes across eight environments.

The effects of processing on parameters directly related to pasta colour, such as both carotenoid pigments and yellow and brown indices, are shown in Figure 3.

Considering the mean genotypic values, decreases of 86.7% of carotenoid pigment content, on average, and 16.0% of the YI were found after processing, while the BI showed an increase of 8.2%. These variations are of the same order of magnitude as those found by Borrelli et al. [12]. The differences in the extent of pigment content and YI variation after pasta making could be due to the different analysis methods used (chemical vs. physical/colorimetric). It should be noted, however, that since the YI is an overall measure of yellowness, its value includes the presence of other yellow carotenoid pigments, even if they are present in small amounts [25].

#### 3.2.2. Multiple Regression Analysis for Pasta Colour

To define the relationship between all traits involved in this study, a simple Pearson correlation analysis was performed. The results are shown in Table 2.

Correlation analysis indicated that YI-S had the highest and positive significant association with YI-P (*r* = 0.93, *p*-value = 0.000), followed by, in decreasing order, β-Car-S (*r* = 0.66, *p*-value = 0.000), Lut-S (*r* = 0.64, *p*-value = 0.000), HP (*r* = 0.26, *p*-value = 0.002), and BI-S (*r* = 0.21, *p*-value = 0.012).

Regarding enzymatic activities, HP did not show any correlation with BL and the POD and PPO enzymes, which instead were correlated with each other.

LOX’s HP and BL activities significantly influenced carotenoid pigment content in pasta in the opposite way. HP showed a positive correlation, while BL showed a negative one with carotenoid pigment, confirming their involvement in the degradation of carotenoids during processing (Table 2). Despite HP and BL being the result of the activity of the same enzyme, different roles for them have been described [44]. LOX catalyses, in the first instance, the hydroperoxidation of polyunsaturated fatty acids (HP), producing free and peroxy radicals. This reaction mainly occurs during mixing, due to the concurrence of a greater availability of oxygen provided with water and of mechanical action that promotes substrate–enzyme–O_2_ contact, other than the modification of surface hydrophobicity, which might result in the release and activation of enzymes and lipids. In the subsequent phases of pasta processing, carotenoid pigment degradation (BL) prevails, and it is due to a coupled oxidation mechanism, mediated by reactive oxygen species originating during fatty acid oxidation [12]. The same was highlighted during bread making, when the greatest decreases in carotenoids in flour were observed during the kneading stage [45]. These different reactions are not closely related. In fact, other than the fact that they seem to act in different phases of the pasta making process [12], LOX isoenzymes could intervene to different extents in pigment degradation [46]. This could also explain the lack of a significant correlation between the two activities observed in semolina (Table 2) and the negative significant correlation observed only between BL activity and both pigments in semolina.

A highly significant correlation was found between PPO and POD activities. Both enzymes were shown to be involved in brownness although with different reaction mechanisms [17,18,19,20,47]. In fact, these enzymes have some common phenolic substrates and other specific ones for each of them, whose oxidation leads to the browning of products [47]. This explains the significant correlation between their activity and BI of semolina and pasta. The different levels of significance observed for correlations with the BI in semolina and pasta could also be due to the nonenzymatic browning caused by the Maillard reaction that takes place during the drying phase of the process (Table 2). This reaction is highly dependent on the amount and type of substrate available (sugars and proteins) and moisture content, as well as the time–temperature combinations of the drying treatment [48,49], although we can assume that the degree of involvement in our study is low because of the low-temperature drying cycle applied.

The high correlation between the BI in semolina and the BI in pasta indicates that pasta brownness would also be dependent on the inherent brownness of semolina as well as pasta processing [17].

Both in semolina and pasta, lutein and β-carotene correlated with each other and with the YI, confirming that they are mainly responsible for the bright yellow colour in semolina and contribute to the commercial value of pasta.

The strength of the relationship between the YI and pigment content or their biosynthetic enzymes was also evidenced by the identification of quantitative trait loci (QTLs) that often co-mapped in the same chromosome region, increasing the effectiveness of marker-assisted selection (MAS) programmes for these traits [39,50,51]. This was also confirmed in other cereal species [52,53,54].

Even if it is reasonable that the YI of semolina should predict the YI of pasta, due to the high correlation (*r* = 0.93) between these traits, previous studies [11,55] demonstrated that pigment losses can occur during pasta making due to LOX activities. Moreover, POD and PPO, influencing BI-P, could negatively affect yellowness [19]. For this reason, the mean values of the G x E interaction were used in a multiple regression model, considering YI-P as the dependent variable and all the parameters analysed in semolina as predictors or regressors or independent variables to select variables with greater weight in predicting YI-P.

For the YI-P regression model, an ANOVA showed a highly significant *F* value (Table 3) with an estimated model error of 0.878. Regarding independent variables, the selection method based on Mallow’s Cp statistic extracted only two variables, which were always present in all proposed subset models, with the first subset having the lowest Cp value (0.790) corresponding to the BI-S (*β*-coefficient = −0.107) and YI-S (*β*-coefficient = 0.962) variables (Table 4). The ANOVA showed, for these two variables, as well as for the intercept, highly significant univariate tests in addition to high values of the coefficient of multiple regression (*R* = 0.932), of determination (*R*^2^ = 0.868), and of correct determination (*R*^2^*_adj_* = 0.866) (Table 3).

The collinearity statistics for the BI-S and YI-S predictors included in the multiple regression model for the YI-P dependent variable showed very favourable values of tolerance, VIF (Variance Inflation Factor), and residual *R*^2^, demonstrating that the two predictors fit the model of regression very well for estimating YI-P (Table 4). This was also supported by the value of the Durbin–Watson statistic (d) for the estimation of residual autocorrelation (Table 4). The *β*-coefficients of the BI-S and YI-S variables included in the multiple regression model contribute 10.7% and 96.2%, respectively, to the YI-P variable, but their behaviour is different since BI-S contributes minimally and negatively to the estimate of YI-P, as compared to YI-S.

These results underline the usefulness of evaluating the YI of semolina, commercially identified as being yellow in colour, as a fast and reliable method for performing a screening for pigment content and, ultimately, for large-scale selection in durum wheat breeding programmes for pasta colour [15,24,25,38,56]. Due to the very high correlation of the YI (Minolta b* index) with the quantity of yellow pigment in semolina measured by chemical extraction and quantified by spectrophotometric and/or by HPLC methods, the use of light reflectance via a Minolta chromameter, requiring no chemicals, could represent a rapid alternative to time-consuming quantification. Previously, it had already been demonstrated that the colour measurement of a small amount of whole meal samples using a scanning NIR instrument correlated well (*r* = from 0.74 to 0.90) with semolina’s b* values measured with a Minolta chromameter, offering a speedy prediction of semolina colour especially in early generation selection of breeding [57,58]. Moreover, since semolina is the raw material used for pasta production, from the point of view of the supply chain, knowing the level of pigments in semolina in the early stages of selection, when its quantity is not enough for pasta pilot plants, is very important for making a prediction of the final pasta colour.

Our results do not agree with previous studies. In fact, Borrelli et al. [11] found that LOX activities were prevalent in influencing the loss of pigments during pasta processing. Furthermore, the identification of the deletion of the *Lpx-B1.1* allele in durum wheat resulting in a 4.5-fold reduction in LOX activity was determined to lead to a significant improvement in pasta colour [55]. This was also confirmed by Fu et al. [59]. This discrepancy could be due to the greater variability in genotypes and environments (each including different locations) investigated, which gives greater strength to the results. The involvement of the BI, although minimal, would seem to indirectly indicate the role of POD and PPO in the final pasta colour.

### 3.3. Principal Component Analysis

**Figure 4:**
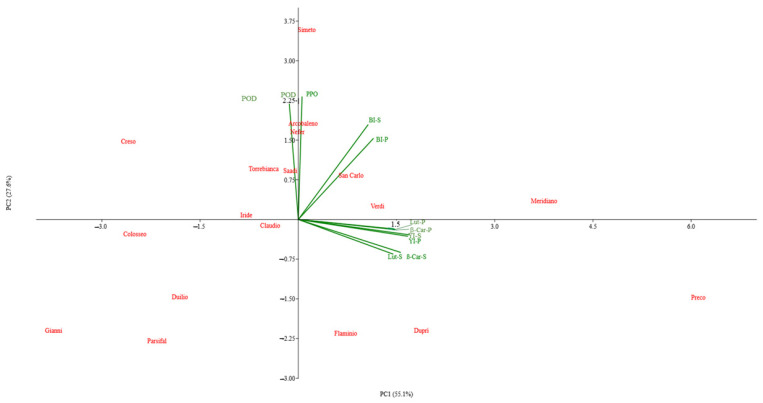
A PCA biplot showing the distribution of the traits analysed in semolina and pasta and of genotypes along the two principal factors. POD = peroxidase activity; PPO = polyphenoloxidase activity; Lut = lutein; β-Car = β-carotene; YI = yellow index; BI = brown index; S = semolina; P = pasta.

To establish a stronger correlation among the traits analysed in the eighteen genotypes, a PCA was performed, and the results are shown in Figure 4.

About 83% of the total variability is explained by the components PC1 (55.1%) and PC2 (27.6%). Three groupings are evident. The first group is significantly and positively associated with PC1 and includes all traits directly related to yellowness, analysed in semolina and pasta, which are highly correlated to each other (Table 2), and it is mainly linked to the genotype Verdi. In the second group, positively associated with PC2, are the two enzymes PPO and POD, mainly responsible for brownness [10,11,12,13], which are strictly related to Arcobaleno, Nefer, Saadi, and Torrebianca. Finally, in the third group, there are BI-S and BI-P that resulted from POD and PPO activities as well as from the level of the grey component due to the accumulation of minerals in grain (ash content) or that is produced during the processing of pasta [10]. The two vectors of BI-S and BI-P are about at 45° with respect to the point of insertion of two axes and, therefore, are not significantly associated with the components PC1 and PC2. Among genotypes, only the San Carlo genotype is aligned on these components. HP and BL were not significantly correlated with any other variable and were not selected in PCA.

For the other genotypes, PC2 clearly distinguishes Simeto and, to a lower extent, Arcobaleno, Nefer, and Saadi, from Flaminio, while Meridiano, as opposed to Colosseo, is well represented by the PC1 component with Iride and Claudio which reflect the same trend with respect to Verdi. The remaining genotypes, almost all in the lower quadrants, are a bit scattered and can be considered isolated cases, with Preco being the most isolated by far.

### 3.4. High-Performance Index (HPI)—Tool for Descriptive Identification of Genotype with Best Combination of Traits Involved in Colour of Pasta

Most of the traits considered in this study showed a strong genotypic component, and each trait played its own role, positive or negative, in determining the pasta colour of each genotype. A descriptive method for determining the relative contribution of each trait and identifying the genotype that reaches the best compromise among all the traits that affect semolina and pasta colour was previously developed by Troccoli et al. [26], using a mathematical algorithm named the High-Performance Index (HPI). In HPI calculations, the enzymatic activities of LOX, POD, and PPO in semolina and the BI in semolina and pasta were considered negative variables for the final colour (lower levels were desirable), while carotenoid content and the YI in semolina and pasta were considered positive variables (higher levels were welcomed). After standardizing the deviation values of each variable with respect to its overall mean value, the algorithm checked whether the variable was specified as negative or positive and, for each deviation, calculated a positive or negative score that varies from 0 to a maximum of 2 as the deviation value increases. The score assigned to each analytical variable represents the specific contribution of that trait and therefore its performance. The partial score of the row-by-column crossing of each cell represents, for each genotype, the Specific Performance Index (SPI) for each variable. The joint use of the HPI and SPI helps to immediately visualize how much each variable (SPI) contributes to the final HPI.

**Figure 5:**
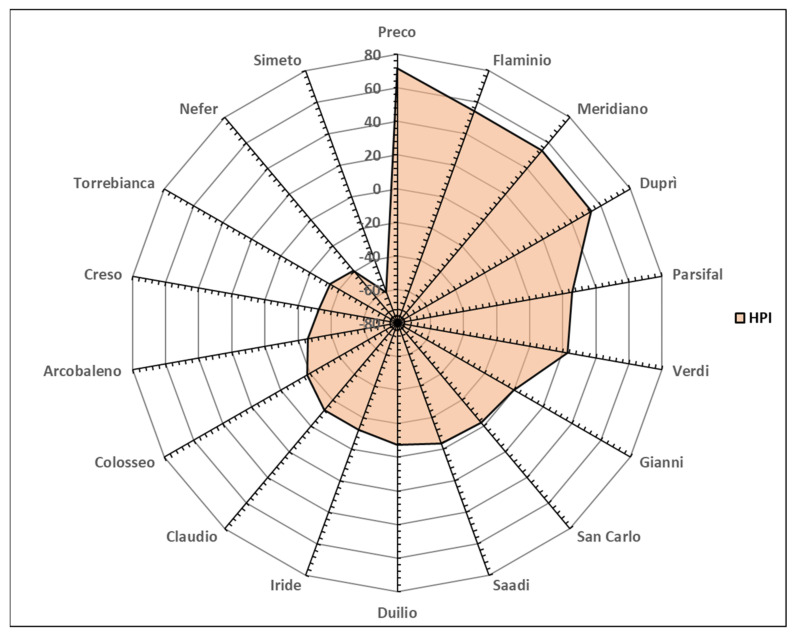
Spider chart for High-Performance Index (HPI) for semolina and pasta of eighteen genotypes grown in eight different Italian environments.

In Table S3, for each genotype, the partial score attributed to each variable is reported, and their sum was used to calculate the HPI. Considering the mean values of the G x E interaction, each of the twelve variables has eight replicate values, one for each environment. Therefore, for each genotype, the maximum expected HPI value is, in absolute value, 192 (2 × 8 Env × 12 Var), if all 12 variables obtain a score of 2 in each environment. The HPI was represented with a radar chart (Figure 5), showing that the best performance was obtained by the Preco genotype which achieved the highest HPI value (71.6), since most of the variables were favourable, presenting a positive SPI (Table S3).

In detail, the Preco genotype reached the maximum negative value (2 × 8 Env) of the SPI for HP, since this trait had the highest value in semolina (Table 1), but this negative characteristic was largely compensated for by the positive SPI values of the other traits, excluding the low values of the BI of semolina and pasta. The Preco genotype was followed by a group of genotypes comprising Flaminio, Meridiano, and Duprì that showed HPI values close to each other (54.3 to 53.3) due to the positive score for most traits. On the contrary, the most negative HPI value was attributed to Simeto, since all the variables had negative SPI scores, due to high enzymatic activities and low pigment contents (Table 1 and Table S3).

The same tool was used to identify the most promising environment for colour expression (Table 5).

A narrower range for the environmental HPI was evidenced as compared with genotype one (Table S3), further confirming the influence of genotype on colour traits. It is interesting to observe that the environment negatively affected both enzyme activities, producing positive SPI scores, and colour-related traits, producing negative scores (Table 4). The environments with the best HPI values were the North-Central Tyrrhenian Coast (ENV3), under sub-littoral conditions, and the North-Central Mountainous Apennines (ENV4), generally characterized by abundant autumn rainfall, a dry winter with low temperatures, and limited rainfall and a high maximum temperature during the grain filling stage until ripening. Insular environments (ENV7 and ENV8), together with the North-Central Adriatic Coast (ENV2), showed intermediate performance. Finally, the South-Central Mountainous Apennines (ENV5), characterized by different rainfall distributions and quantities across locations and lower temperatures during winter and higher ones in the final period of the growth cycle with respect to multi-year seasonal averages [29], had a more negative HPI value and showed a highly negative SPI value for β-Car-P.

However, from a broader perspective, it is necessary to consider the effects of some abiotic stresses (mainly drought, salinity, and high temperatures) on the pigment content of cereal grains [36,60,61,62]. This is even more important when considering the scenario of climate change in which stress conditions are increasingly present and involve larger geographical areas, orienting the choice towards more tolerant genotypes, which are capable of facing emerging challenges and assuring at the same time a good yield and quality performance.

## 4. Conclusions

Knowledge of the relative contribution of genotype, environment, and their interaction to colour expression is very important for setting up appropriate breeding programmes in agreement with the quality requirements of the food market. The results reported in this study confirm the importance of the genotypic component in terms of traits related to colour expression.

For the multiple regression model, we selected semolina’s YI as the key trait to be used in predicting the bright yellow colour of pasta so appreciated by consumers. This model can be considered very robust because it derives from a multi-environmental dataset. Although the data presented come from experimental trials carried out in the past, this feature makes them widely applicable.

The YI is closely linked to the content of carotenoid pigments. During pasta processing, other traits, such as enzymes, intervene to modify the content of carotenoid pigments, even if they seem to contribute marginally to the final pasta colour.

Furthermore, since the analysed traits intervene in different ways in determining colour expression, the HPI could represent an innovative tool to use to simplify data interpretation, as it provides a unique value to express the variability in the effects of each trait. The consistency of the dataset makes this index able to provide reliable indications for making the best choice of genotypes/environments for producing more coloured pasta.

In a future study, it would be interesting to verify these results in more recent genotypes, mostly cultivated along the Italian territory, improved for production and other qualitative characteristics.

## Figures and Tables

**Table 1:** Mean values of traits related to semolina and pasta colour of eighteen genotypes grown in eight Italian environments.

	HP (EU g^−1^)	BL (EU g^−1^)	POD (EU g^−1^)	PPO (EU g^−1^)	Lut-S (µg g^−1^, DM)	ß-Car-S (µg g^−1^, DM)	YI-S	BI-S	Lut-P (µg g^−1^, DM)	ß-Car-P (µg g^−1^, DM)	YI-P	BI-P
Genotype (G; *n* = 24)												
Arcobaleno	1.284 ± 0.427	0.006 ± 0.003	154.492 ± 45.203	273.034 ± 113.304	1.718 ± 0.675	0.485 ± 0.164	23.438 ± 0.748	14.113 ± 1.201	0.165 ± 0.054	0.047 ± 0.018	19.425 ± 1.054	15.438 ± 1.337
Claudio	1.442 ± 0.471	0.010 ± 0.007	118.625 ± 36.392	207.330 ± 51.962	2.148 ± 0.607	0.560 ± 0.16	22.759 ± 0.993	13.500 ± 0.994	0.181 ± 0.06	0.054 ± 0.026	18.450 ± 0.978	14.638 ± 1.116
Colosseo	0.937 ± 0.419	0.005 ± 0.002	138.693 ± 39.417	140.928 ± 46.412	1.031 ± 0.247	0.210 ± 0.211	19.783 ± 0.928	13.213 ± 0.888	0.259 ± 0.085	0.039 ± 0.017	17.775 ± 0.517	14.125 ± 0.747
Creso	0.671 ± 0.428	0.005 ± 0.005	131.700 ± 40.375	256.388 ± 63.158	1.117 ± 0.229	0.177 ± 0.037	18.688 ± 1.174	13.875 ± 1.184	0.124 ± 0.043	0.047 ± 0.018	16.525 ± 1.323	14.838 ± 1.163
Duilio	4.640 ± 1.610	0.004 ± 0.003	15.030 ± 10.055	60.240 ± 31.166	1.092 ± 0.309	0.339 ± 0.094	21.753 ± 0.924	13.413 ± 1.313	0.148 ± 0.024	0.044 ± 0.015	18.450 ± 1.006	14.613 ± 1.237
Duprì	0.921 ± 0.277	0.007 ± 0.004	8.291 ± 3.496	64.113 ± 22.515	2.946 ± 0.716	0.745 ± 0.227	26.324 ± 1.034	13.775 ± 0.905	0.204 ± 0.041	0.068 ± 0.038	21.400 ± 1.143	14.788 ± 0.926
Flaminio	0.970 ± 0.252	0.001 ± 0.001	17.086 ± 5.515	84.943 ± 38.013	2.336 ± 0.495	0.652 ± 0.184	25.441 ± 0.672	13.450 ± 1.250	0.219 ± 0.060	0.046 ± 0.016	21.200 ± 1.089	14.471 ± 1.212
Gianni	0.870 ± 0.151	0.004 ± 0.002	9.318 ± 4.214	61.018 ± 28.986	1.084 ± 0.202	0.170 ± 0.043	18.709 ± 0.893	12.925 ± 0.994	0.135 ± 0.025	0.024 ± 0.014	16.338 ± 1.081	14.000 ± 1.068
Iride	1.052 ± 0.267	0.007 ± 0.003	106.818 ± 30.023	137.060 ± 54.426	1.844 ± 0.351	0.361 ± 0.074	22.889 ± 1.018	13.838 ± 1.034	0.181 ± 0.036	0.027 ± 0.017	19.063 ± 1.068	14.825 ± 1.193
Meridiano	1.312 ± 0.281	0.001 ± 0.003	110.448 ± 37.772	227.706 ± 69.657	2.621 ± 0.658	0.776 ± 0.215	25.670 ± 1.290	14.225 ± 1.004	0.416 ± 0.250	0.157 ± 0.092	21.088 ± 1.339	15.288 ± 1.244
Nefer	0.967 ± 0.331	0.020 ± 0.008	142.755 ± 33.476	268.312 ± 88.313	1.376 ± 0.481	0.344 ± 0.095	23.761 ± 0.739	14.188 ± 0.793	0.236 ± 0.086	0.045 ± 0.023	20.263 ± 0.826	15.150 ± 0.956
Parsifal	0.509 ± 0.217	0.004 ± 0.002	26.010 ± 23.127	96.948 ± 46.591	1.695 ± 0.297	0.379 ± 0.025	20.867 ± 0.772	12.788 ± 0.967	0.192 ± 0.078	0.036 ± 0.016	17.850 ± 0.621	14.050 ± 1.087
Preco	5.056 ± 0.861	0.002 ± 0.002	34.454 ± 10.969	78.097 ± 35.682	2.952 ± 1.002	0.923 ± 0.339	31.482 ± 0.708	14.300 ± 1.181	0.464 ± 0.247	0.145 ± 0.077	25.925 ± 2.177	15.425 ± 1.257
Saadi	0.411 ± 0.231	0.005 ± 0.003	137.448 ± 48.702	171.027 ± 41.246	1.811 ± 0.411	0.473 ± 0.120	22.777 ± 0.794	14.000 ± 0.945	0.160 ± 0.071	0.055 ± 0.031	19.213 ± 1.084	15.275 ± 1.056
San Carlo	1.655 ± 0.665	0.006 ± 0.006	107.216 ± 42.485	285.593 ± 126.047	1.809 ± 0.452	0.705 ± 0.243	24.445 ± 0.783	13.900 ± 0.892	0.204 ± 0.048	0.049 ± 0.02	20.350 ± 1.471	15.238 ± 0.994
Simeto	4.062 ± 1.547	0.007 ± 0.004	167.517 ± 30.868	494.108 ± 146.923	1.711 ± 0.359	0.319 ± 0.071	23.013 ± 1.054	14.575 ± 1.057	0.222 ± 0.075	0.038 ± 0.02	19.388 ± 1.411	15.438 ± 1.056
Torrebianca	4.898 ± 1.315	0.005 ± 0.003	97.698 ± 34.32	246.375 ± 57.289	1.567 ± 0.396	0.339 ± 0.095	22.989 ± 0.706	14.200 ± 0.919	0.221 ± 0.070	0.042 ± 0.014	18.625 ± 0.506	14.863 ± 1.308
Verdi	1.524 ± 1.230	0.005 ± 0.002	51.122 ± 17.611	192.983 ± 46.37	1.356 ± 0.388	0.408 ± 0.132	25.033 ± 1.343	13.713 ± 1.088	0.273 ± 0.076	0.110 ± 0.030	20.563 ± 1.438	15.788 ± 1.183
*F*_(17,119)_	39.014	10.066	58.806	25.122	29.661	34.272	204.441	8.630	8.322	12.666	39.059	7.625
*p*-value	0.000	0.000	0.000	0.000	0.000	0.000	0.000	0.000	0.000	0.000	0.000	0.000
**Environment (E; *n* = 54)**												
ENV1	2.207 ± 1.814	0.007 ± 0.004	88.085 ± 53.558	150.317 ± 91.262	1.939 ± 0.627	0.524 ± 0.239	23.687 ± 2.818	14.894 ± 0.767	0.257 ± 0.108	0.091 ± 0.065	19.711 ± 1.39	16.061 ± 0.854
ENV2	2.536 ± 2.342	0.004 ± 0.003	63.798 ± 44.962	143.255 ± 95.172	1.584 ± 0.537	0.501 ± 0.235	22.418 ± 3.144	13.928 ± 0.541	0.186 ± 0.079	0.071 ± 0.039	18.333 ± 2.457	15.067 ± 0.635
ENV3	2.021 ± 2.121	0.009 ± 0.009	76.794 ± 51.64	230.835 ± 166.08	1.578 ± 0.632	0.504 ± 0.265	22.737 ± 3.093	14.078 ± 0.705	0.159 ± 0.068	0.059 ± 0.030	18.944 ± 2.372	14.989 ± 0.810
ENV4	1.780 ± 1.622	0.003 ± 0.004	74.848 ± 50.823	175.371 ± 127.671	2.258 ± 0.784	0.533 ± 0.286	23.518 ± 2.993	12.744 ± 0.630	0.265 ± 0.079	0.060 ± 0.031	20.228 ± 2.512	13.806 ± 0.569
ENV5	1.722 ± 1.604	0.003 ± 0.003	60.804 ± 41.421	132.449 ± 68.127	2.223 ± 0.759	0.497 ± 0.252	23.304 ± 3.334	13.072 ± 0.604	0.344 ± 0.259	0.082 ± 0.088	20.017 ± 2.651	14.106 ± 0.644
ENV6	1.589 ± 1.312	0.006 ± 0.004	90.043 ± 62.742	208.868 ± 104.753	1.902 ± 0.700	0.520 ± 0.282	22.942 ± 3.145	13.217 ± 0.637	0.218 ± 0.081	0.052 ± 0.035	19.389 ± 2.692	14.361 ± 0.730
ENV7	1.329 ± 1.171	0.007 ± 0.006	106.996 ± 66.394	217.845 ± 142.554	1.892 ± 0.692	0.491 ± 0.207	24.833 ± 2.831	15.406 ± 0.665	0.169 ± 0.083	0.034 ± 0.024	20.511 ± 2.305	16.761 ± 0.787
ENV8	1.564 ± 1.255	0.005 ± 0.005	138.508 ± 84.528	228.261 ± 158.994	0.941 ± 0.517	0.148 ± 0.072	23.146 ± 2.790	12.878 ± 0.701	0.180 ± 0.059	0.029 ± 0.018	19.261 ± 2.055	14.070 ± 0.739
*F*_(7,119)_	5.273	6.661	27.058	7.434	32.645	26.788	27.362	79.108	9.413	9.267	9.500	69.328
*p*-value	0.000	0.000	0.000	0.000	0.000	0.000	0.000	0.000	0.000	0.000	0.000	0.000
**G × E Interaction (*n* = 3)**												
*F*_(119,288)_	249.274	143.747	185.877	144.206	142.449	13.295	8.766	17.325	1593.217	1949.488	21.520	20.032
*p*-value	0.000	0.000	0.000	0.000	0.000	0.000	0.000	0.000	0.000	0.000	0.000	0.000
Overall mean	1.843 ± 1.726	0.006 ± 0.006	87.485 ± 62.699	185.9 ± 128.421	1.79 ± 0.768	0.465 ± 0.266	23.323 ± 3.077	13.777 ± 1.126	0.222 ± 0.132	0.06 ± 0.05	19.549 ± 2.414	14.903 ± 1.216

HP = hydroperoxidation activity of LOX; BL = bleaching activity of LOX; POD = peroxidase activity; PPO = polyphenoloxidase activity; Lut = lutein; β-Car = β-carotene; YI = yellow index; BI = brown index; S = semolina; P = pasta; ENV1 = Po Valley; ENV2 = North-Central Adriatic Coast; ENV3 = North-Central Tyrrhenian Coast; ENV4 = North-Central Mountainous Apennines; ENV5 = South Mountainous Apennines; ENV6 = Adriatic–Ionic; ENV7 = Sicily; ENV8 = Sardinia. Values are reported as mean ± Standard deviations (*n* = 3). For G, E, and G × E factors, number of data (*n*) used for mean value is shown in brackets: for G: *n* = 24 corresponds to 3 replicates (rep) × 8 environments; for E: *n* = 54 corresponds to 3 rep × 18 genotypes; for G × E interaction, *n* = 3 corresponds to rep considered for analysis.

**Table 2:** Pearson’s correlation matrix and *p*-values for all traits involved in colour expression.

	HP	BL	POD	PPO	Lut-S	ß-Car-S	YI-S	BI-S	Lut-P	ß-Car-P	BI-P
**BL**	−0.10										
	*p = 0.239*										
**POD**	−0.12	**0.34**									
	*p = 0.165*	***p = 0.000***									
**PPO**	0.09	**0.37**	**0.71**								
	*p = 0.265*	***p = 0.000***	***p = 0.000***								
**Lut-S**	0.05	**−0.23**	**−0.27**	**−0.20**							
	*p = 0.585*	***p = 0.006***	***p = 0.001***	***p = 0.019***							
**ß-Car-S**	0.13	**−0.18**	**−0.30**	**−0.17**	**0.86**						
	*p = 0.118*	***p = 0.033***	***p = 0.000***	***p = 0.036***	***p = 0.00***						
**YI-S**	**0.30**	−0.07	−0.14	−0.06	**0.66**	**0.71**					
	***p = 0.000***	*p = 0.389*	*p = 0.085*	*p = 0.513*	***p = 0.00***	***p = 0.00***					
**BI-S**	**0.19**	**0.32**	**0.24**	**0.28**	0.09	**0.23**	**0.33**				
	***p = 0.022***	***p = 0.000***	***p = 0.003***	***p = 0.001***	*p = 0.261*	***p = 0.007***	***p = 0.000***				
**Lut-P**	**0.19**	**−0.23**	−0.12	−0.10	**0.50**	**0.43**	**0.50**	−0.03			
	***p = 0.019***	***p = 0.006***	*p = 0.147*	*p = 0.246*	***p = 0.000***	***p = 0.000***	***p = 0.000***	*p = 0.681*			
**ß-Car-P**	**0.19**	**−0.21**	**−0.19**	−0.14	**0.50**	**0.57**	**0.50**	0.16	**0.80**		
	***p = 0.026***	***p = 0.013***	***p = 0.022***	*p = 0.091*	***p = 0.000***	***p = 0.000***	***p = 0.000***	*p = 0.052*	***p = 0.00***		
**BI-P**	0.10	**0.26**	**0.21**	**0.21**	0.06	**0.22**	**0.35**	**0.93**	−0.04	**0.19**	
	*p = 0.229*	***p = 0.002***	***p = 0.013***	***p = 0.010***	*p = 0.440*	***p = 0.009***	***p = 0.000***	***p = 0.00***	*p = 0.635*	***p = 0.026***	
**YI-P**	**0.26**	−0.08	−0.14	−0.09	**0.64**	**0.66**	**0.93**	**0.21**	**0.53**	**0.45**	**0.23**
	***p = 0.002***	*p = 0.354*	*p = 0.095*	*p = 0.286*	***p = 0.000***	***p = 0.00***	***p = 0.00***	***p = 0.012***	***p = 0.000***	***p = 0.000***	***p = 0.005***

HP = hydroperoxidation activity of LOX; BL = bleaching activity of LOX; POD = peroxidase activity; PPO = polyphenoloxidase activity; Lut = lutein; β-Car = β-carotene; YI = yellow index; BI = brown index; S = semolina; P = pasta. The means of the G × E interaction of the traits (*n* = 144) were used for analysis. The values of significant correlation coefficients are in bold.

**Table 3:** ANOVA for regression model (summary).

Dependent Variable	Source of Variation	df	Sum of Square	Mean Square	*F*	*p*-Value	Statistics	Value
**YI-P**	**Regression**	2	715.281	357.641	464.089	0.000	*R*	0.932
	**Error**	141	108.659	0.771			*R* ^2^	0.868
	**Total**	143	823.940				*R* ^2^_adj_	0.866
							Error of Model	0.878
	**A univariate significance test for the YI-P variable of the subset of the best predictors chosen according to the Mallow Cp procedure**							
	***Intercept***	1	23.156	23.156	30.048	0.000		
	***BI-S***	1	8.481	8.481	11.005	0.001		
	***YI-S***	1	679.191	679.191	881.346	0.000		
	Error	141	108.659	0.771				

**Table 4:** Collinearity statistics for predictors included in regression model for YI-P dependent variable.

Predictors IN	Tolerance	VIF (Variance Inflation Factor)	*R* ^2^	*B*-Coefficient	*β*-Coefficient	Partial Correlation	Semi-Partial Correlation	*t*	*p*-Value
**BI-S**	0.891	1.122	0.109	−0.231	−0.107	−0.269	−0.101	−3.317	0.001
**YI-S**	0.891	1.122	0.109	0.752	0.962	0.928	0.908	29.687	0.000
**Durbin–Watson statistic (d) test for residual autocorrelation in the regression model (*n* = 144)**									
	**d lower tabulated**	**d value**	**d upper tabulated**		**Serial correlation**				
	1.468	1.723	1.767		0.137				

YI = yellow index; BI = brown index; S = semolina; P = pasta.

**Table 5:** For each environment, the cumulative score (Specific Performance Index) of the eighteen genotypes, attributed to each analytical variable, was calculated using the HPI tool, according to the procedure described in Troccoli et al. [19]. For each environment, the HPI is the sum of the score of the 12 variables.

Specific Performance Index (SPI; *n* = 18) for Negative (NV *) or Positive (PV *) Variable													
Specific Attributes	NV	NV	NV	NV	PV	PV	PV	NV	PV	PV	PV	NV	HPI
Environment	HP	BL	POD	PPO	Lut-S	β-Car-S	YI-S	BI-S	Lut-P	β-Car-P	YI-P	BI-P	
ENV3	7.03	4.93	−0.03	2.00	−0.48	−0.35	−0.23	−0.20	−0.90	−0.58	−0.20	−0.2	10.80
ENV4	4.83	6.13	0.12	1.73	−0.20	−0.63	−0.23	−0.23	−0.23	−0.78	−0.18	−0.25	10.10
ENV7	4.38	3.78	−0.08	1.75	−0.20	−0.20	−0.18	−0.23	−0.70	−1.68	−0.23	−0.25	6.17
ENV2	4.75	2.63	0.60	1.88	−0.30	−0.28	−0.20	−0.18	−1.08	−1.50	−0.20	−0.2	5.92
ENV8	3.38	5.00	−0.23	1.30	−1.15	−1.10	−0.20	−0.28	−0.25	−1.13	−0.30	−0.175	4.88
ENV6	3.30	1.95	−0.08	0.02	−0.23	−1.05	−0.18	−0.23	−0.25	−2.35	−0.18	−0.275	0.47
ENV1	2.98	0.33	−0.20	1.38	−0.18	−0.23	−0.23	−0.23	−0.55	−2.88	−0.20	−0.25	−0.25
ENV5	4.55	3.78	0.18	−0.15	−0.25	−0.30	−0.23	−0.25	−4.23	−7.53	−0.23	−0.225	−4.88

HP = hydroperoxidation activity of LOX; BL = bleaching activity of LOX; POD = peroxidase activity; PPO = polyphenoloxidase activity; Lut = lutein; β-Car = β-carotene; YI = yellow index; BI = brown index; S = semolina; P= pasta; ENV1: Po Valley; ENV2: North-Central Adriatic Coast; ENV3: North-Central Tyrrhenian Coast; ENV4: North-Central Apennines; ENV5: Central Mountainous Apennines (South); ENV6: Adriatic–Ionic; ENV7: Sicily; ENV8: Sardinia. * A variable classified as “negative” (NV) or “positive” (PV) is considered favourable if the initial content of specific variable results respectively lower or higher than the overall mean value, obtaining a positive score.

## Data Availability

The original contributions presented in this study are included in the article/Supplementary Materials. Further inquiries can be directed to the corresponding author.

## Supplementary Materials

The following supporting information can be downloaded at https://www.mdpi.com/article/10.3390/foods14030392/s1, Table S1: Year of release, country, and pedigree information of eighteen durum wheat genotypes grown in 1999–2000 crop years in eight climate areas; Table S2: Variance components for all traits measured in semolina and pasta of eighteen durum wheat genotypes grown in eight environments; Table S3: For each genotype, the cumulative score (Specific Performance Index) of the eight environments attributed to each analytical variable was calculated using the HPI tool, according to the procedure described in Troccoli et al. [19]. For each genotype, the HPI is the sum of the score of the 12 variables.
