# Supplementary material for: Prediction of Pasta Colour Considering Traits Involved in Colour Expression of Durum Wheat Semolina
Source: Foods. 2025 Jan 24;14(3):392. doi: 10.3390/foods14030392 (PMC11817165; doi:10.3390/foods14030392)
Supplement: Supplementary file 1 [file foods-14-00392-s001.zip › Table S3.pdf]

Table S3. For each genotype, the cumulative score (Specific Performance Index) of the eight environments attributed to each analytical variable was calculated using the HPI tool, according to the procedure described in Troccoli et al. [19]. For each genotype, the HPI is the sum of the score of the 12 variables.

	Specific Performance Index (SPI; *n*=8) for Negative (NV*) or Positive (PV*) Variable												HPI
**Specific Attributes**	**NV**	**NV**	**NV**	**NV**	**PV**	**PV**	**PV**	**NV**	**PV**	**PV**	**PV**	**NV**	
**Variety**	**HP**	**BL**	**POD**	**PPO**	**Lut-S**	**β-Car-S**	**YI-S**	**BI-S**	**Lut-P**	**β-Car-P**	**YI-P**	**BI-P**	
**Preco**	-16.0	11.1	9.4	9.1	9.3	12.3	5.5	-0.7	12.6	14.4	5.1	-0.6	71.6
**Flaminio**	7.4	13.1	12.8	8.7	5.0	6.4	1.4	0.3	0.0	-2.3	1.3	0.4	54.3
**Meridiano**	4.4	12.7	-4.2	-3.9	7.5	10.5	1.5	-0.6	9.9	15.8	1.2	-0.5	54.1
**Duprì**	7.6	-2.3	14.4	10.3	9.9	9.2	2.0	-0.1	-1.1	2.0	1.5	0.0	53.3
**Parsifal**	11.6	5.0	11.6	7.5	-0.3	-1.6	-1.8	1.1	-1.8	-4.8	-1.5	0.8	25.7
**Verdi**	3.8	2.6	6.7	-1.0	-4.1	-2.4	1.1	-0.1	3.8	12.7	0.7	-1.0	22.7
**Gianni**	8.2	3.7	14.2	10.6	-6.3	-10.1	-3.3	0.9	-6.1	-10.0	-2.7	0.9	0.1
**San Carlo**	1.3	2.8	-3.3	-7.3	-0.1	7.3	0.7	-0.3	-0.8	-2.8	0.5	-0.5	-2.4
**Saadi**	12.0	0.2	-8.8	1.1	0.2	0.4	-0.5	-0.4	-4.7	-2.2	-0.4	-0.6	-3.7
**Duilio**	-14.8	5.2	13.4	10.4	-6.5	-4.4	-1.2	0.4	-5.1	-4.2	-1.0	0.3	-7.6
**Iride**	6.5	-4.0	-3.8	4.3	0.6	-3.0	-0.4	-0.2	-2.8	-9.1	-0.5	-0.1	-12.3
**Claudio**	3.5	-8.1	-5.9	-1.9	3.0	2.9	-0.5	0.2	-3.2	-1.6	-1.0	0.2	-12.3
**Colosseo**	7.6	1.3	-9.5	3.5	-6.9	-8.8	-2.6	0.6	2.5	-4.9	-1.6	0.7	-18.1
**Arcobaleno**	4.0	-0.4	-11.9	-7.1	-1.5	-0.2	0.0	-0.5	-4.3	-3.3	-0.2	-0.7	-26.0
**Creso**	10.5	3.1	-8.1	-5.9	-5.9	-9.6	-3.3	-0.2	-7.3	-3.3	-2.6	0.0	-32.5
**Torrebianca**	-16.0	1.9	-1.7	-5.3	-2.2	-4.4	-0.3	-0.6	-0.4	-3.9	-0.9	-0.1	-33.7
**Nefer**	7.2	-16.0	-10.8	-7.2	-4.3	-4.3	0.2	-0.6	0.7	-4.5	0.5	-0.4	-39.5
**Simeto**	-13.5	-3.3	-14.2	-15.9	-0.4	-4.5	-0.3	-1.0	-0.3	-6.3	-0.3	-0.7	-60.6
	**Number of varieties with favourable SPI ***												
**Favourable SPI**	14	12	7	9	7	7	7	6	6	4	7	8	7
**Unfavourable SPI**	4	6	11	9	11	11	11	12	12	14	11	10	11

HP=Hydroperoxidation activity of LOX; BL=Bleaching activity of LOX; POD=Peroxidase activity; PPO=Polyphenoloxidase activity; Lut=Lutein; β-Car=β-Carotene; YI=Yellow index; BI=Brown index; S=semolina; P= Pasta.

*A variable classified as "negative" (NV) or "positive" (PV) is considered favourable if the initial content of specific variable results respectively lower or higher than the overall mean value, obtaining a positive score.
