# Supplementary material for: Prediction of Pasta Colour Considering Traits Involved in Colour Expression of Durum Wheat Semolina
Source: Foods. 2025 Jan 24;14(3):392. doi: 10.3390/foods14030392 (PMC11817165; doi:10.3390/foods14030392)
Supplement: Supplementary file 1 [file foods-14-00392-s001.zip › Table S1.pdf]

Table S1. Year of release, country and pedigree information of the eighteen durum wheat genotypes grown in the 1999-2000 crop years in the eight climate areas.

Genotype				
**Accession**	**Year of Release**	**Country**	**Pedigree**	**Climate Areas**
Creso	1974	Italy	Yaktana-54/Norin 10-B//2*Cappelli-63/3/3*Tehuacan-60/4/Capelli-B144	ENV1 – Po Valley (5)*
Duilio	1984	Italy	Cappelli//Anhinga/Flamingo	ENV2 – North-Central Adriatic Coast (8)
Simeto	1988	Italy	Capeit-8/Valnova	ENV3 – Central Thyrrhenian Coast (6)
Gianni	1992	Italy	Multiple cross among durum wheat cultivar	ENV4 – Central Mountainous Apennines (6)
Parsifal	1992	France	INRA 92-1/D81028	ENV5 – South Mountainous Apennines (7)
Arcobaleno	1995	Italy/Spain	Chen/Altar 84	ENV6 –Adriatic-Ionic (4)
Colosseo	1995	Italy	Mutant Mexa/Creso	ENV7 – Sicily (8)
Preco	1995	Italy	Edmore/WPB881//Selected line 3	ENV8 – Sardinia (6)
Iride	1996	Italy	Altar 84/Ares	
Nefer	1996	France	164/Keops	
San Carlo	1996	Italy	Grazia/Degamit	
Claudio	1998	Italy	CIMMYT's selection 35/Durango//IS1938/Grazia	
Duprì	1998	Italy	Duilio/Primadur	
Flaminio	1998	Italy	Latino/Cappelli	
Saadi	1998	Italy/France	IDSM72-3/711.8	
Verdi	1998	Italy/France	Valdur/Regal/Mexicalli	
Meridiano	1999	Italy	Simeto/WB 881/Duilio/F21	
Torrebianca	1999	Italy	Creso/Trinakria	

* In brackets are the number of locations considered for each climate area.
