# Supplementary material for: Prediction of Pasta Colour Considering Traits Involved in Colour Expression of Durum Wheat Semolina
Source: Foods. 2025 Jan 24;14(3):392. doi: 10.3390/foods14030392 (PMC11817165; doi:10.3390/foods14030392)
Supplement: Supplementary file 1 [file foods-14-00392-s001.zip › Table S2.pdf]

Table S2. Variance components for all traits measured in semolina and pasta of the eighteen durum wheat genotypes grown in the eight environments.

Source of Variation	HP		BL		POD		PPO		Lut-S		ß-Car-S		YI-S		BI-S		Lut-P		ß-Car-P		YI-P		BI-P	
	**REV**	**(% of Total)**	**REV**	**(% of Total)**	**REV**	**(% of Total)**	**REV**	**(% of Total)**	**REV**	**(% of Total)**	**REV**	**(% of Total)**	**REV**	**(% of Total)**	**REV**	**(% of Total)**	**REV**	**(% of Total)**	**REV**	**(% of Total)**	**REV**	**(% of Total)**	**REV**	**(% of Total)**
**Genotype (G)**	2.481	79.2	0.000	46.0	3116.016	74.6	11904.788	68.7	0.355	56.4	0.046	61.7	9.060	90.4	0.208	14.9	0.007	38.4	0.001	50.0	4.614	75.2	0.235	14.5
**Environment (E)**	0.124	4.0	0.000	12.8	624.300	14.9	1411.249	8.1	0.174	27.7	0.016	21.2	0.522	5.2	0.945	67.7	0.004	19.6	0.000	15.7	0.458	7.5	1.078	66.3
**GxE Interaction**	0.520	16.6	0.000	40.3	428.921	10.3	3920.779	22.6	0.098	15.6	0.010	13.7	0.316	3.2	0.205	14.7	0.008	41.9	0.001	34.2	0.925	15.1	0.270	16.6
**Error**	0.006	0.2	0.000	0.8	6.960	0.2	82.136	0.5	0.002	0.3	0.003	3.3	0.122	1.2	0.038	2.7	0.000	0.1	0.000	0.1	0.135	2.2	0.043	2.6
**TOTAL**	**3.13**		**0.00**		**4176.20**		**17318.95**		**0.63**		**0.08**		**10.02**		**1.40**		**0.02**		**0.00**		**6.13**		**1.63**	

REV=Relative Estimated Variance. HP=Hydroperoxidation activity of LOX; BL=Bleaching activity of LOX; POD=Peroxidase activity; PPO=Polyphenoloxidase activity; YI=Yellow Index; BI=Brown Index; Lut=Lutein; β-Car=β-Carotene; YI=Yellow Index; BI=Brown Index; S=Semolina; P=Pasta.
